# Nucleolar localization of influenza A NS1: striking differences between mammalian and avian cells

**DOI:** 10.1186/1743-422X-7-63

**Published:** 2010-03-17

**Authors:** Romain Volmer, Beryl Mazel-Sanchez, Christelle Volmer, Sébastien M Soubies, Jean-Luc Guérin

**Affiliations:** 1 INRA, UMR 1225, Ecole nationale vétérinaire de Toulouse, F-31076 Toulouse, France; 2 Université de Toulouse; ENVT; UMR 1225; F-31076 Toulouse, France; 3 Centre for Biomolecular Sciences, School of Biology, University of St. Andrews, North Haugh, St. Andrews, Fife KY16 9ST, UK

## Abstract

In mammalian cells, nucleolar localization of influenza A NS1 requires the presence of a C-terminal nucleolar localization signal. This nucleolar localization signal is present only in certain strains of influenza A viruses. Therefore, only certain NS1 accumulate in the nucleolus of mammalian cells. In contrast, we show that all NS1 tested in this study accumulated in the nucleolus of avian cells even in the absence of the above described C-terminal nucleolar localization signal. Thus, nucleolar localization of NS1 in avian cells appears to rely on a different nucleolar localization signal that is more conserved among influenza virus strains.

## Findings

The nucleolus is a highly dynamic multifunctional subnuclear compartment [1]. It is the site of ribosomal RNA synthesis and ribosomal subunits assembly. In addition, the nucleolus is increasingly recognized as a critical regulator of many other cellular functions, including the regulation of mitosis, cell growth and response to stress [1-3]. The nucleolus is also emerging as an important target of various viral proteins [4]. Viral proteins targeting the nucleolus are for example implicated in the regulation of apoptosis, as shown with West Nile virus capsid protein, and in the regulation of viral mRNA export, as shown with human immunodeficiency virus Rev protein and with herpesvirus saimiri ORF57 protein [5-7]. However, for most viruses, consequences of viral protein localization in the nucleolus remain largely unknown [3,4].

The non-structural 1 (NS1) protein of influenza A viruses NS1 is a multifunctional protein, known to interact with and modify the function of many cellular proteins, thereby creating a cellular environment favouring virus replication [8]. Recently, a nucleolar localization signal (NoLS) has been identified in NS1 [9]. This NoLS targets NS1 to the nucleolus of mammalian cells. Presently, the role of the nucleolar localization of NS1 in the viral cycle is unknown. One can speculate that NS1 proteins targeting the nucleolus of mammalian cells could modify the functions of nucleolar proteins. The mammalian NoLS of NS1 consists of a stretch of C-terminal basic amino acids that are present only in certain strains of influenza A viruses [9]. Thus, only certain NS1 proteins accumulate in the nucleolus of mammalian cells. Whether NS1 proteins accumulate in the nucleolus of avian cells is currently unknown.

In this study, we compared the nucleolar localization of NS1 of different influenza virus strains in mammalian and avian cells using immunocytochemistry and confocal microscopy. Experiments were done in human A549 alveolar epithelial cells and in primary embryonic fibroblasts used between passages 2 and 6, cultured from 11 days old Balb/c mouse (*Mus musculus*) embryos, from 14 days old Pekin duck (*Anas platyrhynchos*) embryos or from 12 days old chicken (*Gallus gallus*) embryos. Cells were infected at a multiplicity of infection (MOI) of 3 plaque forming units (pfu) per cell (MOI = 3) with the human influenza A/Udorn/72(H3N2) strain (designated Udorn), the human laboratory adapted influenza A/PR/8/34(H1N1) strain (designated PR8), the avian influenza A/Turkey/Italy/977/V99(H7N1) strain (designated 977) or the avian influenza A/Turkey/Italy/4426/00(H7N1) strain (designated 4426). At 3, 4, 6, 8 and 12 hours post-infection (hpi), cells were fixed with 4% Paraformaldehyde, permeabilized with Phosphate Buffered Saline (PBS) 0.5% Triton X-100 and incubated for one hour in PBS 0.1% Triton X-100 and 2% Bovine Serum Albumin. Antibody incubation was performed overnight at 4°C.

**Figure 1:**
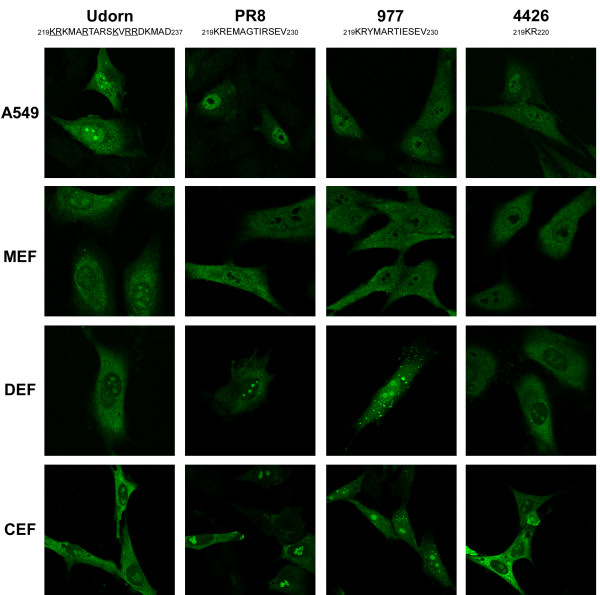
**Subcellular localization of NS1 in infected cells**. Human A549 alveolar epithelial cells, mouse embryonic fibroblasts (MEF), duck embryonic fibroblasts (DEF) and chicken embryonic fibroblasts (CEF) were infected at a MOI = 3 with different strains of influenza virus. The cells were fixed, stained with a rabbit anti-NS1 polyclonal antibody and a secondary FITC-labelled anti-rabbit antibody and imaged with a confocal microscope. Shown are representative pictures obtained from cells fixed 4 hpi. The C-terminal amino acid sequence of NS1 is indicated under the name of each viral strain. The basic amino acids identified by Melen *et al. *as defining the mammalian NoLS are present in the NS1 of Udorn and are underlined.

The C-terminal sequence of Udorn NS1 protein contains the basic amino acids identified by Melen et al. as defining the mammalian NoLS (underlined in Figure 1), whereas the other NS1 proteins lack one or more of these basic amino acids [9]. Consequently, only the NS1 of Udorn accumulated in the nucleolus of primary mouse embryonic fibroblasts (MEF) and of A549 human respiratory cells (Figure 1). NS1 proteins of the other viruses tested did not accumulate in the nucleolus of mammalian cells irrespective of the time post-infection (Figure 1). By contrast, the NS1 of all viruses used in this study accumulated in the nucleolus of primary duck embryonic fibroblasts (DEF) and primary chicken embryonic fibroblasts (CEF) at 4 hpi (Figure 1). Thus, all NS1 proteins tested have an amino acid sequence forming a functional NoLS in avian cells. In addition, our results show that the amino acids required to target NS1 to the nucleolus of avian cells differ from the amino acids required to target NS1 to the nucleolus of mammalian cells.

**Figure 2:**
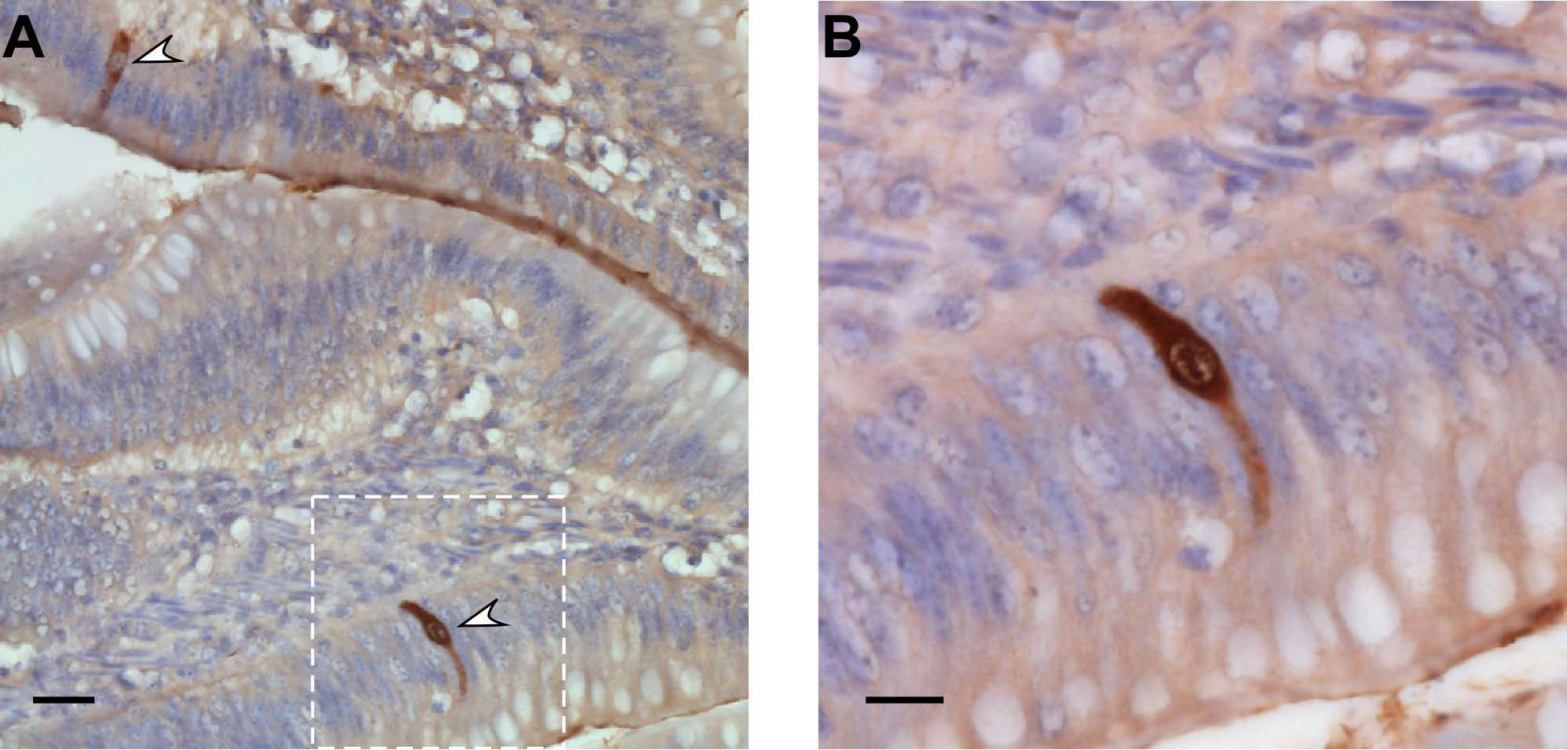
**Subcellular localization of NS1 in duck ileal epithelial cells *in vivo***. A) Ileum collected 6 days post-infection from a Pekin duck infected orally with 10^7 ^pfu of the 977 virus, formalin fixed, sectioned at 3 μm. Immunohistochemical anti-NS1 staining detected with a peroxidase-coupled secondary antibody revealed with diaminobenzidine, hematoxylin counterstained. Arrowheads point to NS1 positive enterocytes. Scale bar = 20 μm. B) Magnification of the dotted region shown in panel A. NS1 staining is detected in the cytoplasm and in subnuclear structures, corresponding to nucleoli. Scale bar = 10 μm.

Then, we verified that NS1 targets the nucleolus *in vivo*. We infected two-week old Pekin ducks orally with 10^7 ^pfu of the 977 virus. Previous experiments performed with this virus had shown that the ileum and the colon were the major sites of virus replication (our unpublished observation). Immunohistochemical staining of 3 μm paraffin embedded ileal sections with a rabbit polyclonal anti-NS1 antibody (Figure 2) revealed the presence of viral antigens in enterocytes 6 days post-infection. Anti-NS1 antibodies detected with a peroxidase-coupled secondary antibody revealed with diaminobenzidine stained the cytoplasm and subnuclear structures, corresponding to nucleoli (Figure 2). Thus, the subcellular localization of NS1 *in vitro *is consistent with its nucleolar localization in duck intestinal epithelial cells.

**Figure 3:**
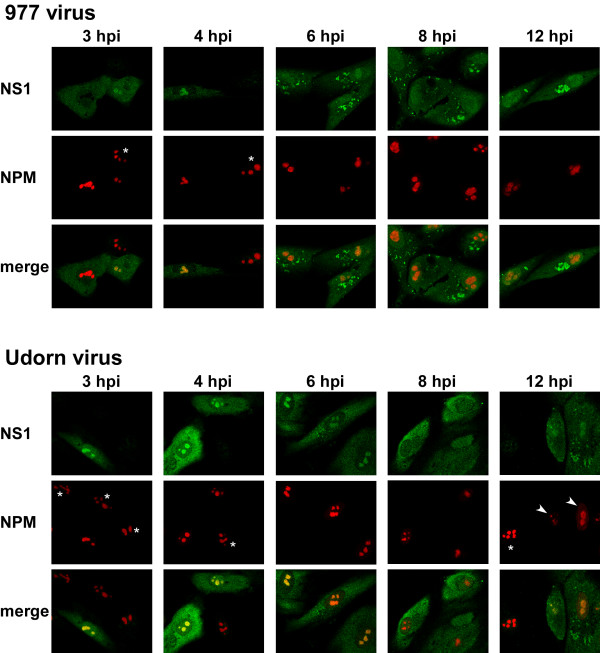
**Time course analysis of NS1 and NPM staining in DEF infected with the 977 or the Udorn virus**. DEF were infected at a MOI = 3 with the 977 or the Udorn virus and fixed at the indicated time post-infection. Cells were stained with a rabbit anti-NS1 polyclonal antibody and a mouse anti-NPM monoclonal antibody, revealed with a secondary FITC-labelled anti-rabbit antibody and a secondary RhodamineX-labelled anti-mouse antibody. FITC and RhodamineX fluorescences were acquired sequentially on a confocal microscope. Asterisks point to the NPM staining pattern in non-infected cells. Arrowheads point to virus-induced changes in NPM staining pattern.

Viral infections can lead to changes in the nucleolar morphology, likely caused by virus-induced disruption of nucleolar functions, as shown for the infectious bronchitis coronavirus and for the herpes simplex virus 1 [10,11]. We therefore analyzed whether nucleolar localization of NS1 modified the expression pattern of nucleophosmin (NPM), a nucleolar protein that localizes to the granular centre of the nucleolus [3]. We performed a time course analysis of the intracellular localization of NS1 and NPM (Figure 3) in DEF infected at a MOI = 3 with either the 977 or the Udorn viruses. In DEF, NS1 of both viruses colocalized with NPM (Figure 3). Nucleolar localization of NS1 was visible 3 hpi and was maximal between 4 and 6 hpi (Figure 3). Nucleolar accumulation declined starting 6 hpi. The intensity of nucleolar NS1 staining eventually became indistinguishable from the nucleoplasmic NS1 staining between 8 and 12 hpi. In addition, we detected bright cytoplasmic foci of NS1 in DEF infected with the 977 virus (Figure 1&3). These foci were reminiscent of previously described virus-induced cytoplasmic inclusions that remain of uncertain identity [12]. No apparent change in the pattern of NPM expression was observed in DEF infected with the 977 virus. By contrast, starting 12 hpi, faint NPM staining could be detected in the nucleoplasm of Udorn infected cells, suggesting that a fraction of NPM is displaced from the nucleolus to the nucleoplasm following infection. Increased levels of NPM in the nucleoplasm, as well as ring-like NPM staining pattern were detected in about 50% of Udorn infected cells at 12 hpi. Interestingly, changes in NPM staining pattern has also been observed following infection with the coronavirus infectious bronchitis virus whose nucleocapsid protein targets the nucleolus [10]. In DEF infected with Udorn, changes in the nucleolar morphology appeared between 8 and 12 hpi, corresponding to a stage in the virus life cycle where cytopathic effects, such as membrane blebbing became visible (date not shown). Thus, rather than being due to a direct effect of NS1 on nucleolar functions, disruption of the nucleolar morphology in influenza virus infected cells could result from virus induced intracellular stress. Alternatively, displacement of NPM from the nucleolus to the nucleoplasm could be due to an interaction of the viral ribonucleoprotein complex with NPM, as shown in MDCK cells infected with the influenza A/WSN/33 virus [13].

Presently the role of the nucleolar localization of NS1 in influenza virus cycle is unknown. In mammalian cells, nucleolar accumulation of NS1 occurs only with certain strains of influenza A viruses. As the NS1 of all viruses studied here targeted the nucleolus of avian cell, we speculate that the nucleolar localization of NS1 could be an important step during the viral cycle in avian cells. Whether, nucleolar localization of NS1 contributes to virulence is currently unknown. Valuable information would certainly be obtained by studying the phenotype of a reverse genetics engineered virus lacking a functional NoLS. In order to perform such studies in avian cells, the avian NoLS needs to be identified. Our results show that the avian NoLS relies on an amino acid sequence that is present in all the influenza virus strains tested in this study, and thus could be conserved among most influenza virus strains.

## Competing interests

The authors declare that they have no competing interests.

## Authors' contributions

Conceived and designed the experiments: RV. Performed the experiments: RV, BMS, CV, SMS. Analyzed the data: RV, BMS, JLG. Wrote the paper: RV, BMS. All authors read and approved the final manuscript.
